# Understanding the glacial methane cycle

**DOI:** 10.1038/ncomms14383

**Published:** 2017-02-21

**Authors:** Peter O. Hopcroft, Paul J. Valdes, Fiona M. O'Connor, Jed O. Kaplan, David J. Beerling

**Affiliations:** 1 Bristol Research Initiative for the Dynamic Global Environment, School of Geographical Sciences and Cabot Institute, University of Bristol, University Road, Bristol BS8 1SS, UK; 2 Met Office Hadley Centre, FitzRoy Road, Exeter EX1 3PB, UK; 3 Institute of Earth Surface Dynamics, University of Lausanne, Geopolis Building, CH-1015 Lausanne, Switzerland; 4 Department of Animal and Plant Sciences, Alfred Denny Building, University of Sheffield, Sheffield S10 2TN, UK

## Abstract

Atmospheric methane (CH_4_) varied with climate during the Quaternary, rising from a concentration of 375 p.p.b.v. during the last glacial maximum (LGM) 21,000 years ago, to 680 p.p.b.v. at the beginning of the industrial revolution. However, the causes of this increase remain unclear; proposed hypotheses rely on fluctuations in either the magnitude of CH_4_ sources or CH_4_ atmospheric lifetime, or both. Here we use an Earth System model to provide a comprehensive assessment of these competing hypotheses, including estimates of uncertainty. We show that in this model, the global LGM CH_4_ source was reduced by 28–46%, and the lifetime increased by 2–8%, with a best-estimate LGM CH_4_ concentration of 463–480 p.p.b.v. Simulating the observed LGM concentration requires a 46–49% reduction in sources, indicating that we cannot reconcile the observed amplitude. This highlights the need for better understanding of the effects of low CO_2_ and cooler climate on wetlands and other natural CH_4_ sources.

## Abstract (web summary)

The cause of the increase in atmospheric methane from 375 p.p.b.v. during the last ice age to 680 p.p.b.v. at the onset of Industrialization remains uncertain. Here, using an Earth system model, the authors show that we cannot reconcile this rise based on our current understanding of natural methane sources.

Methane (CH_4_) is a potent greenhouse gas and it plays a fundamental role in atmospheric chemistry, regulating the oxidizing capacity of the troposphere and influencing surface concentrations of ozone (O_3_), carbon monoxide (CO) and volatile organic compounds (VOCS) including biogenic VOCs (BVOCs). The atmospheric concentration of CH_4_ has risen sharply from a concentration averaged over AD 800–1600 of 680 p.p.b.v. (parts per billion volume) characterizing the late Holocene pre-industrial era^1^,^2^ to 1,799 p.p.b.v. by the year 2010 (ref. 3). The increase in concentration contributed around 17% of the radiative forcing from well-mixed greenhouse gases since AD1750 (ref. 4). While the cause of increasing atmospheric CH_4_ concentrations over the industrial era is unequivocally a result of anthropogenic activities, variability in CH_4_ over recent decades, including a stabilization of concentrations, is not fully understood^3^, because of persistent uncertainties in both anthropogenic and natural processes^5^. Renewed growth in atmospheric CH_4_ since 2007 is likely driven by biogenic sources, highlighting the importance of natural and/or agricultural sources of methane^6^.

The dominant natural source of CH_4_ is wetlands, which constitute around 60–80% of natural emissions and around one third of the total flux to the atmosphere at present^3^. During the last glacial maximum (LGM), two processes may have led to a reduction in wetland CH_4_ sources: (1) a reduction in wetland area, particularly in the high latitudes of both hemispheres and (2) the cooler and generally drier climate, and low atmospheric carbon dioxide (CO_2_) concentrations, which led to reduced rates of methanogenesis. While large ice-sheets prevented wetland formation in North America and Northern Europe, the 120 m drop in sea-level exposed large regions of continental shelf suitable for wetland formation, in particular around Indonesia (Sunda Shelf) and between Australia and New Guinea (Arafura Shelf). Sea-level effects might offset the reduced emissions due to the other factors^7^, but most wetland schemes predict a significant reduction in emissions of between 23 and 67% relative to the pre-industrial (refs 8, 9, 10, 11).

Reduced temperatures and humidity levels will also decrease the rate of removal of CH_4_ from the atmosphere by its primary sink, that is, through reaction with the hydroxyl (OH) radical. Climate affects the rate of formation of OH by photolysis and the reaction rate of OH and CH_4_ (refs 12, 13). The colder and drier LGM atmosphere probably had a reduced OH burden as compared with warmer, more humid interglacial periods. A reduction in OH burden thus implies an increase in CH_4_ lifetime, which could lead, paradoxically, to increased CH_4_ concentrations in a LGM atmosphere. However, CH_4_ is not the only chemical species that is destroyed by OH, and reductions in the emissions of other reactive trace gases could increase the amount of OH available for CH_4_ oxidation. Potential expansion of tropical forests on to exposed continental shelves notwithstanding, the colder, drier climate and low atmospheric CO_2_ of the LGM may have led to substantial reductions in the primary productivity of the terrestrial biosphere. This reduction could have lowered emissions of BVOCs, such as isoprene, particularly from tropical and subtropical forests^8^. A reduction in BVOC emissions could have increased OH, and hence decreased the lifetime of CH_4_. This second effect should act to offset some of the lifetime increase brought about by the overall reduction in OH burden. Thus the balance between the lifetime perturbations associated with BVOCs and physical climate is critical for understanding the LGM CH_4_ lifetime, as it is for the future^13^.

Two previous studies found between 50 and 100% of the reduction in atmospheric CH_4_ at the LGM was caused by a decrease in the CH_4_ lifetime arising in part from reduced BVOC emissions^8^,^14^. More recently it has been proposed that the net result of the climate effects and reduced BVOC emissions on OH and hence CH_4_ lifetime would be negligible, so that most if not all of the glacial reduction in CH_4_ concentration should be attributed to reductions in sources^15^. Changes in emissions of nitrogen oxides (NO_x_) from soils and lightning, and changes in photolysis rates do not alter this conclusion^16^. However, these past studies may have overestimated the reduction in emissions of BVOCs during the LGM, because they did not include the leaf-level stimulation of isoprene emissions caused by the low (185 ppmv) LGM atmospheric CO_2_, which potentially counters the effects from a cooler climate^17^,^18^.

Here we simulate the late Holocene pre-industrial (pre-industrial in the following) and LGM atmospheric CH_4_ cycles using the Earth System model HadGEM2-ES^19^ (see ‘Methods' section). Here pre-industrial refers to the climate state immediately prior to Industrial Revolution around AD1750 (ref. 20). HadGEM2-ES incorporates an interactive CH_4_ cycle based on schemes for dynamic vegetation and wetlands^21^ and interactive tropospheric chemistry^22^. We incorporate a near-complete inventory of the major natural CH_4_ sources (wetlands, peatlands, biomass burning, oceans, termites and geological/hydrates) and atmospheric processes and sinks (for example, oxidation/loss by OH and soil uptake), including aspects which affect CH_4_ lifetime (for example, BVOC emissions and lightning and soil NO_x_ production). To address aspects of stratospheric chemistry, we also include separate sensitivity tests examining O_3_ photolysis and stratospheric O_3_.

Despite a lack of process-based understanding of geological, freshwater and animal CH_4_ emissions, this approach is the most comprehensive yet applied to the glacial CH_4_ cycle. We integrate source and sink processes together within an Earth System model, and include for the first time, estimates of peatland emissions and termite emissions, together with the CO_2_ inhibition of plant isoprene emissions. We include different LGM estimates of wildfire emissions, and therefore address uncertainty in this component. We also compare the main drivers of the change in CH_4_ lifetime.

We find that the Earth System model simulations do not reproduce the observed LGM CH_4_ concentration, overestimating it by around 100 p.p.b.v. There is good agreement with past studies in terms of CH_4_ lifetime change at the LGM, and together with significant uncertainties surrounding natural CH_4_ emissions, this suggests that models of CH_4_ sources are under-sensitive. If confirmed with other modelling approaches, this would have implications for understanding atmospheric CH_4_ change on different timescales.

## Results

### Emissions calculations

**Figure 1:**
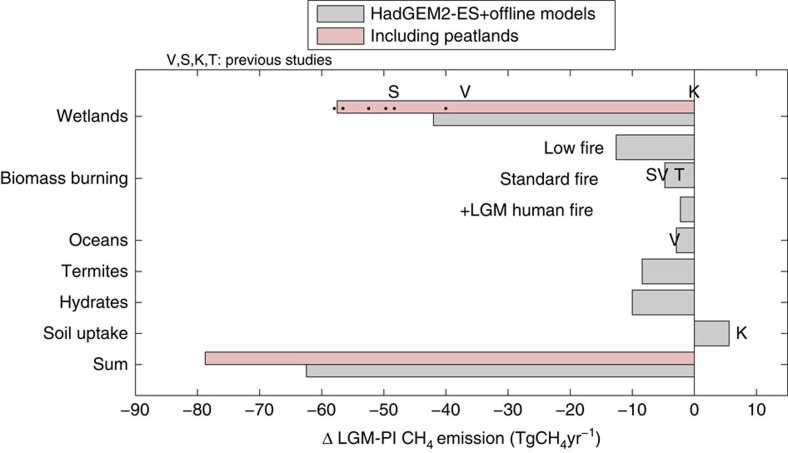
Last glacial maximum minus pre-industrial CH_4_ emissions. Emissions change (TgCH_4_yr^−1^) for each source term and total. Values in pink include peatland emissions in northern extra-tropics. The sum uses the standard-fire flux. Values from previous work have been scaled to the same pre-industrial fluxes as used in our study for comparison. Wetland emissions from ref. 9 (dots) based on PMIP2 coupled GCM simulations, V: Valdes *et al*.^8^, K: Kaplan *et al*.^7^,^14^, T: Thonicke *et al*.^29^ and S: Singarayer *et al*.^10^. More details are provided in Table 1.

The simulated LGM CH_4_ emissions budget is shown in Fig. 1. This compares the predicted changes in emissions from wetlands, biomass burning, oceans, hydrates and termites, as well as bacterial uptake of methane in dry soils. Simulated wetland CH_4_ emissions at the LGM are 70% of the pre-industrial (that is, a reduction from 138 to 97 TgCH_4_yr^−1^). Previous work shows large uncertainty in wetland emissions at the LGM, from no change^7^ to reductions similar to those we simulate^8^,^9^,^10^. The wetland scheme in HadGEM2-ES accounts for changes in soil carbon as a substrate for methanogenesis^21^,^23^ and also the impacts of low LGM atmospheric CO_2_ on the terrestrial carbon cycling. However, in common with the majority of other wetland models^24^,^25^, it does not have a specific representation of hydrological and biogeochemical processes in organic soils or several other peatland-specific processes such as soil water-table variations. We addressed this issue by performing offline simulations using the LPJ-WHyMe model of peatlands and permafrost^26^ forced with HadGEM2-ES climate variables and the atmospheric CO_2_ for each time period. With this model, the high-latitude distribution of wetland emissions is increased, possibly improving on the underestimation of wetland regions in this area under modern climate conditions^27^, and LGM emissions reduce from 70 to 58% of pre-industrial. This enhanced sensitivity is due to the higher proportion of the global emissions deriving from northern latitudes in the peatland model (44 TgCH_4_yr^−1^ versus 16 CH_4_TgCH_4_yr^−1^ in HadGEM2-ES), where additional ice-sheets and enhanced cooling strongly reduce emissions at the LGM. Peatland processes could therefore be important for understanding wetland emissions under LGM conditions. Given the differences in sensitivity of the peatland model, it also highlights the potential for different responses in a future warmer climate.

We simulated biomass burning CH_4_ emissions with LPJ-LMfire^28^ forced offline with HadGEM2-ES climate variables and atmospheric CO_2_. This model shows a 34% reduction (from 14 to 9.2 TgCH_4_yr^−1^), consistent with the range in previous work^8^,^29^. This is termed standard-fire in the following. Inclusion of an estimated human contribution to fires at the LGM gives higher LGM fire emissions, so that the reduction in emissions at the LGM is somewhat smaller at 16% (that is, LGM emissions of 12.1 TgCH_4_yr^−1^), as shown in Fig. 1. This is termed standard+LGM humans. We also include a low-fire scenario as used in previous work^16^ in which all fire emissions are reduced by 90% from pre-industrial values at the LGM. This is included to bracket potential uncertainty in this term, because charcoal-based inferences imply substantial reductions in global biomass burning during the late glacial period^30^. In this scenario, the total fire CH_4_ source term reduces by a factor of 10 (from 14 to 1.4 TgCH_4_yr^−1^). The additional effects due to changes in other trace gas emissions from biomass burning on CH_4_ lifetime are evaluated in the following section.

For the CH_4_ flux from oceans the Guenther *et al*. BVOC scheme^31^ simulates a 20% reduction in BVOC emissions between the pre-industrial and LGM. This is mostly driven by cooler sea-surface temperatures, with a small contribution from reduced surface dissolved organic carbon concentrations. To estimate emissions of CH_4_ from termites, we used the observed relationships between vegetation biome type, termite species and termite CH_4_ emissions per unit biomass to predict termite emissions as a function of vegetation coverage (see ‘Methods' section). We calculated a 40% reduction at the LGM, mostly resulting from reduced tropical forest coverage. In the absence of appropriate process-based schemes for predicting hydrate and other geological CH_4_ emissions, these sources are either assumed to remain unchanged at pre-industrial levels during the LGM, or alternatively were set to zero for the LGM.

Overall, the strength of the total CH_4_ source is reduced by 32–46, 28–42 and 26–41% in the low-fire, standard and standard+LGM human fire scenarios, respectively, where the range depends on whether the peatland model is used and whether emissions from hydrates are reduced (Table 1).

Using the offline models for BVOC, CO and NO_x_ emissions (as described in ‘Methods' section and in the Supplementary Table 1), we find relative changes that are similar to the simulated changes in CH_4_ emissions. The fluxes of CO, acetone and NO_x_ from biomass burning are reduced by 32%, 34% and 30%, respectively, or by 21%, 15% and 7% when LGM human emissions are included. Soil NO_x_ emissions increase by 37%. This is partly as a result of increased LGM land area in the tropics. Transitions from dry to wetter months stimulate emissions in the soil NO_x_ scheme, and a general increase in the precipitation variability across the tropics stimulates NO_x_ emissions in the LGM simulation. The ocean CO flux is reduced by 20%. While acetone emitted from vegetation is reduced by 34%, isoprene emissions are reduced by only 19%, significantly smaller than reductions of up to 31–61%^8^,^14^ in previous work. This is because the stimulation of emission by low CO_2_ during the LGM was not accounted for in these past studies. Lightning NO_x_ is modelled interactively within HadGEM2-ES (see ‘Methods' section). There is a 25% reduction in this source, from 6.1 TgNyr^−1^ in the pre-industrial simulation to 4.6 TgNyr^−1^ for the LGM.

### Factors influencing the glacial CH_4_ lifetime

We ran HadGEM2-ES using the CH_4_ source terms summarized in Fig. 1, other trace gas emissions and a soil uptake term for CH_4_, all described in Table 1 and Supplementary Table 1. In this model, the source trace gases are mostly monthly globally gridded inputs, whereas wetland CH_4_ and lightning NO_x_ are calculated interactively, as are the main sinks of CH_4_ by tropospheric OH oxidation and stratospheric loss (see Supplementary Note 1 for details).

The emissions-driven model predicts a global mean concentration of 659 pbv for the pre-industrial close to the observed AD 800–1,600 average value of 680 p.p.b.v.^1^. The lifetime of CH_4_ with respect to OH is calculated using mixing ratios for CH_4_ and OH up to the diagnosed model tropopause^22^ and is equal to 10.4 years, within the range inferred for present day^32^, and close to the multi-model mean lifetime 10.1±1.7 years for AD1850 conditions found in the Atmospheric Chemistry and Climate Model Intercomparison Project^33^. For the LGM, the lowest LGM concentration is expected in the low-fire LGM scenario, and so this is the only emissions-driven LGM scenario integrated to equilibrium. This results in a concentration of 447 p.p.b.v., which is almost 75 p.p.b.v. greater than the observed LGM concentration of 375 p.p.b.v.

Given that the emissions-driven model over-predicts the LGM CH_4_ concentration, the model-derived methane lifetime will be too long given the self-feedback effect^34^. The self-feedback effect is the positive feedback whereby any change in CH_4_ concentration will influence the amount of OH available for CH_4_ oxidation, which in turn further impacts on the concentration of CH_4_. By running concentration-driven rather than emissions-driven simulations for the two time periods (pre-industrial and LGM), the change in CH_4_ lifetime can be modelled more realistically.

**Figure 2:**
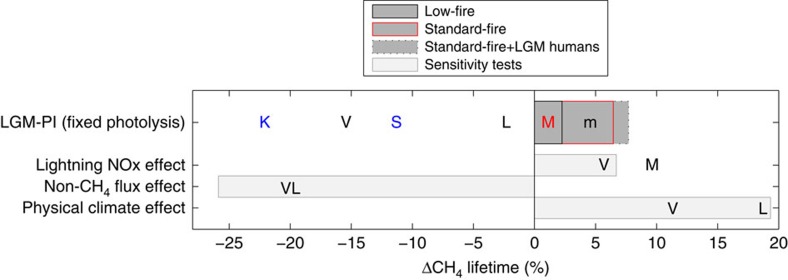
Last glacial maximum atmospheric CH_4_ lifetime change relative to the pre-industrial (%) and estimates of the separate contributions to this change. LGM minus pre-industrial relative CH_4_ lifetime change (dark upper bar), and sensitivity experiments (perturbation minus LGM), light bars below. V: Valdes *et al*.^8^, K: Kaplan *et al*.^7^,^14^, L: Levine *et al*.^15^, S: Singarayer *et al*.^10^ and Murray *et al*.^16^, for which only warm-LGM simulations are included and where m denotes the low-fire scenario. The effects of physical climate changes on reaction rates were not accounted for by Kaplan *et al*.^14^ or by Singarayer *et al*.^10^ shown in blue. Note for the BVOC sensitivity tests, Valdes *et al*.^8^ only changed isoprene and terpene emissions, Levine *et al*.^15^ also changed CO, ethane, propane and acetone; while here soil NO_x_ was also included. Details of all of the lifetime results are given in Table 2.

In the concentration-driven simulations, the LGM lifetime increases by 2.3% relative to the pre-industrial value of 10.4 years for the low-fire scenario, and 6.5% for the standard-fire scenario as shown in Fig. 2. When the standard+LGM human fire scenario is used, the lifetime difference increases to 7.7% (see Table 2). The lifetime differences (LGM-PI) between the three fire scenarios are comparable to that reported in previous work^16^.

In each of the three fire cases, three main factors influence the CH_4_ lifetime: reduced atmospheric temperatures and humidity levels, changes in surface emission rates of CO, NO_x_, isoprene and acetone, and a reduction in the lightning generation of NO_x_ as simulated within HadGEM2-ES. Physical climate changes can be summarized by a global mean cooling of 4.8 °C and a global reduction in water vapour of 24% at the surface, with similar humidity reductions throughout the atmospheric column. The other tropospheric O_3_ precursor emission changes are summarized above, with full details in the ‘Methods' section.

We quantified the relative influences on the CH_4_ lifetime with three additional LGM sensitivity tests with the low-fire interactive CH_4_ (that is, emissions-driven) set-up. The three additional emissions-driven simulations were LGM but with pre-industrial tropospheric O_3_ precursor emissions excluding methane, LGM but with pre-industrial lightning NO_x_ generation rates and LGM but with pre-industrial physical climate. We find that LGM non-CH_4_ emissions cause a 26% decrease in lifetime at the LGM. The LGM lightning reduction is responsible for a lifetime increase of 7%, while the physical climate changes increase the lifetime by 19%. These components of the total lifetime change are shown in Fig. 2. They are comparable with previous work^8^,^15^,^16^ though the stronger cooling in HadGEM2-ES compared with the previous model simulations, is likely responsible for the larger climate term.

For computational efficiency HadGEM2-ES uses pre-computed photolysis rates from an offline 2D model^35^. We also evaluate a model version including an interactive photolysis scheme^36^, which responds dynamically to changes in clouds and aerosols within HadGEM2-ES. This allows the effects from changes in stratospheric O_3_ concentrations on incoming radiation incident on the troposphere, and hence on the production of OH, to be included. Switching from prescribed photolysis rates to interactive photolysis rates together with a prescribed increase in stratospheric ozone^16^ has a very small impact on the methane lifetime, as detailed in the Supplementary Note 2.

### CH_4_ concentration sensitivity to source and lifetime changes

We now combine the model predicted estimates of source and lifetime changes to derive a possible range of concentration predictions for the LGM. Because of the high computational cost of the coupled HadGEM2-ES model, we use a simplified mass balance formulation (described in ‘Methods' section) to calculate the concentration as a function of the global CH_4_ source and the CH_4_ lifetime, and assuming steady state conditions in the two time periods, though this may not hold for all of the late Holocene pre-industrial period^37^. In terms of the sources, we incorporate all of the different source possibilities, including peatlands, changes to hydrates and the three fire scenarios described previously. For lifetime, we apply three different possible changes which correspond to the simulated changes in CH_4_ lifetime in response to the three fire scenarios. We consider changes in sources and sinks separately and then combinations of the two, to give a summary of how the LGM CH_4_ budget depends on the processes analysed. This analysis follows a similar approach recently presented for glacial CO_2_ (ref. 38).

**Figure 3:**
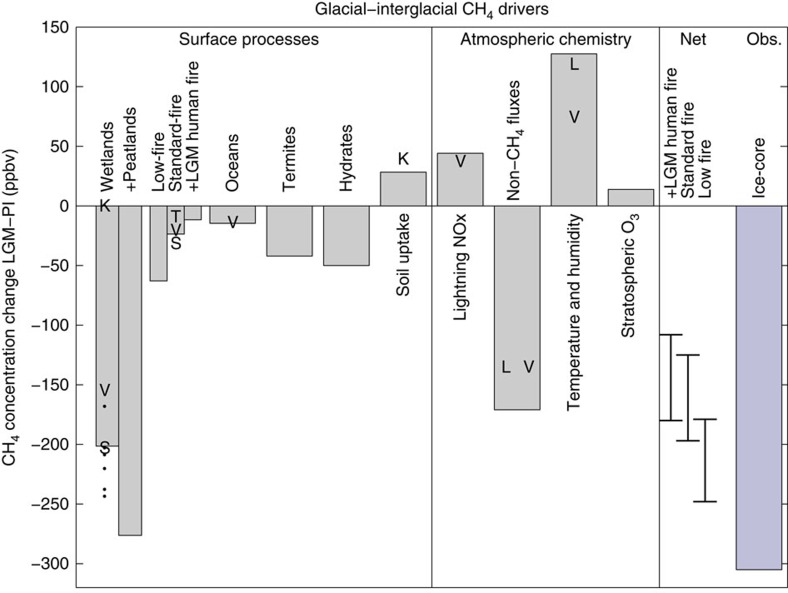
Summary of the contributions to the glacial to inter-glacial CH_4_ budget as modelled in this work and in previous studies. V: Valdes *et al*.^8^, K: Kaplan *et al*.^7^,^14^, L: Levine *et al*.^15^. T: Thonicke *et al*.^29^ and S: Singarayer *et al*.^10^. The contributions from individual sources are calculated assuming no change in lifetime, while the contributions from sinks are calculated with constant source terms. The total concentration changes take account of both source and sink changes. The separate contributions to the change in chemical lifetime of CH_4_ are quantified for the low-fire scenario here. The non-CH_4_ term will be smaller for the standard fire and standard+LGM humans fire scenarios. The uncertainty range in each of the ‘Net' values, encompasses whether or not peatlands are considered. Details of these calculations are given in Supplementary Table 2.

The individual contributions of the source and sink terms considered in this work are shown for comparison in Fig. 3. Our analysis shows that the largest changes in CH_4_ concentrations in absolute terms arise from changes in either the wetland source or tropospheric O_3_ precursors including BVOCs, justifying their focus in previous work. However, the combined contribution from other terms is significant, especially for the sinks. In past work, only relatively few of these terms have been quantified. The most studied aspect is the LGM wetland source for which previous work shows a significant spread, which encompasses the difference between the wetland and peatland models used here.

With a constant CH_4_ lifetime, the wetland and low-fire sources are the largest terms in the PI-LGM amplitude. The other fire terms are significantly smaller. Changes in the lifetime given a constant source are generally larger contributors than many of the individual CH_4_ source terms. The net effect of the lifetime change is small though, because it comprises a combination of several large values of opposing signs.

A successful simulation of the LGM to pre-industrial concentration change must reproduce both the pre-industrial concentration value and the change in concentration between the pre-industrial and LGM, which is 305 p.p.b.v., as shown in Fig. 3. This ice-core inferred amplitude is associated with negligible uncertainty, so we concentrate on model uncertainty in the following. The first criterion is nearly satisfied in this model, as the pre-industrial concentration is 660 p.p.b.v. about 20 p.p.b.v. lower than observed for the period 800–1,600AD. However, a positive trend during this period may result from pre-industrial era anthropogenic activities^1^. Our simulations do not include any anthropogenic sources, and so the 20 p.p.b.v. under-estimate for the pre-industrial in the model is consistent with such a human contribution during this time period. However, uncertainties in each of the source and sink terms as further discussed below, preclude confidence in the attribution of the 20 p.p.b.v. difference between the simulation and observations.

We now assess whether imposing the predicted changes in CH_4_ sources and CH_4_ lifetime (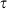) can reproduce the second of the ice-core derived criteria described above. The net effect is estimated by combining for each of the three fire scenarios, the four source estimates (hydrates changing or not, and including peatlands or not) with the respective fire-scenario lifetime change. This gives four concentrations for each of the three scenarios.

In the three fire scenarios, the total CH_4_ LGM source reduction required to give the observed LGM concentration can be calculated with the mass balance formulation (see ‘Methods' section). This results in required source reductions of 46%, 48% or 49% for the low-fire, standard-fire and standard+LGM human fire scenarios, respectively. This range is at the extreme end of our source predictions, which showed maximum reductions of 46%, 42% and 41% for the same three fire scenarios, respectively. These reductions are only 41%, 36% and 34% if the hydrate term is not reduced at the LGM.

The estimated concentration change assuming no change in hydrate emissions is shown in Fig. 3 (and details of the calculations are listed in Supplementary Table 2). For either of the process-based fire estimates (standard or with LGM humans), the resultant CH_4_ concentration predictions all fall short of the observed amplitude at 125–196 p.p.b.v. and 108–181 p.p.b.v. for the standard fire and standard fire with LGM human scenarios, respectively. The range depends on whether peatland model is used or not, with larger changes when the peatland model is used. The low-fire model has both the largest decrease in the CH_4_ source, and the smallest increase in lifetime at the LGM. The modelled PI-LGM concentration change is nearly consistent with the observations in the low-fire scenario when taking the very upper range of the predicted 179–248 p.p.b.v. change.

Conditional on the pre-industrial hydrates flux of 10 TgCH_4_yr^−1^ reducing to zero at the LGM then the predicted concentration changes are larger, at 169–240, 152–224 and 221–290 p.p.b.v. for the standard fire, standard with LGM humans and low-fire scenarios, respectively. We note that the low-fire scenario is unlikely to be consistent with the isotopic record of δ^13^CH_4_, because biomass burning is a source of ^13^C enriched CH_4_, and a significant reduction in biomass burning emissions during the LGM will lead to a depletion in atmospheric *δ*^13^CH_4_ relative to the pre-industrial. This is opposite to the observed 6‰ (permille) enrichment^39^. For a best estimate, we therefore leave out the low-fire scenario, assume hydrate emissions did not change at the LGM (there is little evidence to support a complete shutdown of this source) and include the peatland model. This leads to a calculated LGM concentration of between 463–480 p.p.b.v. for the standard and standard with LGM human fire scenarios. This is 89–105 p.p.b.v. higher than observed for the LGM.

Uncertainty in the source mix for the pre-industrial describes the incomplete knowledge of the relative contributions of different natural (and anthropogenic) CH_4_ sources in the present day and pre-industrial^3^. This is especially important for non-wetland sources such as freshwater, wild animals and geological sources^3^, (see Supplementary Table 3 and Supplementary Note 3). The range of concentrations that arises from this source of uncertainty is assessed in Supplementary Note 3. In these scenarios, we do not include any change in the geological or wild animal sources, but we assume that freshwater emissions scale with wetland emissions. Future work will need to consider recent process-based models of freshwater emissions (ref. 40) to address this assumption. We calculate a maximum difference of 30 p.p.b.v. between three different predictions of the LGM CH_4_ concentration. This is therefore comparable to the range induced by the LGM lifetime changes. However, it is not currently possible to robustly discern between the different source scenarios.

## Discussion

Several aspects of the current understanding of the global CH_4_ cycle are subject to uncertainty. We highlight some key areas that are relevant to the LGM CH_4_ budget and relate them to the results summarized in Fig. 3.

We demonstrate that inclusion of peatland processes is essential for realistically capturing the climatic response. Several other processes are potentially important but require further detailed investigation in this context. These include wetland-specific plant functional types^26^,^41^, seasonal flooding by river systems^41^, ground water controls on wetland formation^42^, nutrient status^43^ and CO_2_ effects on CH_4_ emissions from different types of wetlands^44^. Future work is required to evaluate the relative importance of these processes, though new observations of the CH_4_ cycle may well be required^25^.

Fire is an important natural source of CH_4_, BVOCs, CO and NO_x_. Based on charcoal assemblages, wildfires were thought to be very strongly reduced during the glacial period^30^, although there are relatively few sites covering the LGM. Model predictions are less extreme, showing only a 20–40% reduction in emissions. Because fire affects both the CH_4_ source and the lifetime, we explored the potential influence of biomass burning CH_4_, by including a low-fire scenario in our simulations, but further work is required. The estimated influence of humans on fires during the LGM is non-negligible, but the associated uncertainties are difficult to quantify at present.

Modern day CH_4_ emissions derived from wild animals, geological sources and freshwater bodies have high relative uncertainty of ∼100% around central estimates^3^. Our additional source scenarios (Supplementary Table 3) account for some of this uncertainty by including two alternative estimates for the relative magnitude of the pre-industrial CH_4_ sources. We find that the composition of natural CH_4_ sources that we specify is an important factor for the LGM budget, because large sources that are not directly affected by climate (for example, wild animals or geological) would pose problems for satisfying the LGM CH_4_ budget. Our results imply that large geological and wild animal sources of CH_4_ are not compatible with the presented LGM CH_4_ budget.

The reduced lightning NO_x_ flux plays a major role in the chemical lifetime of CH_4_ at the LGM, increasing it by around 10% and hence offsetting much of the decrease in sources. However, the response of atmospheric convection and lightning to global climate change is highly uncertain^45^. HadGEM2-ES reproduces some aspects of the enhanced dust cycle for the LGM^46^, as inferred from sedimentary archives^47^. However, a hypothesized link between aerosol particles, cloud microphysics and lightning frequency^48^ is not parameterized, but could conceivably have modulated lightning NO_x_ emissions during the LGM.

Large uncertainties surround the prediction of BVOC emissions as a function of climate and ambient CO_2_ (ref. 49). One recent study predicted a 15% increase in isoprene emissions at the LGM^18^ with an empirically defined parameterization of the CO_2_ suppression effect. This contrasts with the 19% reduction found here. A 15% increase in BVOC emissions would mean even larger CH_4_ emissions reductions are required during the LGM relative to the pre-industrial. These results imply there are major uncertainties in the sensitivity of isoprene emissions to climate state. Recent progress in process-based understanding may provide new insight into BVOC emissions responses to past climates^49^,^50^ as it relates synthesis of isoprene to excess electron transport over that required for photosynthesis within the leaf. The advantage of this is that it removes the need for separate empirical parameterisations of the influence of different environmental controls of BVOC emissions as have been used to date.

The short-wave cloud forcing for the LGM-PI has been shown for this model previously^51^. Despite marked changes over tropical land areas, we find a relatively limited effect via photolysis rates on the LGM CH_4_ lifetime in HadGEM2-ES. The new version of the Hadley Centre model, HadGEM3 includes a prognostic as opposed to diagnostic cloud scheme^52^ and this shows much higher cloud cover fractions than HadGEM2-ES. This will likely alter the predicted response of photolysis rates when this newer physical model is coupled with an interactive chemistry scheme. There is also uncertainty in the radiative forcing from stratospheric ozone in response to elevated CO_2_ in climate chemistry model simulations^53^,^54^, that may be important for understanding model dependency in oxidizing capacity related to stratospheric chemistry processes.

In conclusion, Quaternary fluctuations in greenhouse gas concentrations can serve as fundamental tests for understanding of the Earth System. The CH_4_ cycle is particularly interesting because it is also highly sensitive to abrupt climate change with rapid rises of up to 200 p.p.b.v. over a few decades during Dansgaard–Oeschger events of the glacial period^55^. CH_4_ is also an important greenhouse gas, and as such it is imperative to improve our understanding of the feedbacks between climate and natural CH_4_ emissions, and the LGM is a potentially valuable example, because it is likely that emissions were substantially reduced.

We used an Earth System model and a series of offline models of trace gas emissions from natural sources to quantify the primary controls on the atmospheric CH_4_ cycle during the last glacial maximum relative to the pre-industrial. Our comprehensive modelling framework incorporates several relatively untested processes, including peatland CH_4_ emissions, CO_2_ inhibition of isoprene and a process-based representation of natural and anthropogenic biomass burning. However, the modelled best-estimate LGM CH_4_ concentration is between 463 and 480 p.p.b.v., which significantly overestimates the observed concentration of 375 p.p.b.v.

There is relatively good agreement between previous work and results reported here for the relative influence of climate, BVOCs and lightning on the LGM CH_4_ lifetime. There is also relatively limited lifetime change simulated for the LGM, and this implicates a major reduction of CH_4_ sources during the LGM^15^,^16^. Uncertainty in CH_4_ sources also appears to outweigh that in the sink processes, because of the large inter-model uncertainty in wetland CH_4_ schemes^25^ and the large uncertainty surrounding estimates for other natural sources^3^. This is somewhat different to the situation for the historical and future studies where uncertainty in the lifetime is important.

Current CH_4_ emission models appear to underestimate the change needed to balance the LGM budget. This could be attributed either to incorrect parameterization of processes in existing models, or to missing processes, as outlined above. An enhanced sensitivity of CH_4_ sources would be important, as it could alter our understanding of climatically driven variations in atmospheric CH_4_ concentration, such as during rapid rises of the last glacial^55^ and possibly during recent decades and the future.

Further work is required to systematically identify whether factors including groundwater control on wetland formation, wetland-specific carbon cycling or others listed, will change the environmental sensitivity of these aspects of the CH_4_ cycle. Future work could benefit considerably from a formalized probabilistic analysis of this problem, following a similar approach taken for aerosols^56^. This would allow uncertainties in all of the model components to be propagated through to final predictions, allowing an objective assessment of whether the observations are reconciled by the model, and potentially highlighting key uncertainties in a more systematic and objective manner.

## Methods

### Earth system model

We use a coupled atmosphere-vegetation-chemistry model HadGEM2-ES^19^ to analyse the LGM to pre-industrial atmospheric CH_4_ increase. HadGEM2-ES is a comprehensive Earth System model, and consists of a 3D ocean general circulation model (GCM) coupled to a 3D atmospheric GCM, although here we utilize an atmosphere-only configuration. The atmosphere has a horizontal resolution of 1.875° × 1.25° with 38 unequally spaced vertical levels reaching to over 39 km. The model simulates interactive dynamic vegetation^57^, dynamic wetlands and wetland CH_4_ emissions^21^. It includes a tropospheric chemistry scheme from the United Kingdom Chemistry and Aerosols (UKCA) model^22^, which can be coupled with the dynamic wetland emissions scheme. The comprehensive aerosol scheme includes mineral dust (using six tracers) which is coupled to the dynamic vegetation and land surface schemes^19^. HadGEM2-ES does not include stratospheric halogen chemistry^22^, and so ozone mixing ratios from three levels above the dynamically defined tropopause are prescribed based on an observationally based zonally averaged climatology^58^.

Here HadGEM2-ES is coupled to the TropIsop configuration of UKCA^22^, which includes isoprene chemistry missing in the standard CMIP5 version of HadGEM2-ES^19^,^58^. TropIsop employs the lumped Mainz Isoprene Mechanism^22^,^59^, but is otherwise identical to that implemented in HadGEM2-ES for CMIP5 (ref. 58). The model includes 54 chemical species of which 37 are model tracers. It simulates 35 photolytic reactions, 114 bimolecular reactions and 15 uni- and termolecular reactions^22^. Photolysis rates are pre-calculated using an offline 2D model^35^ with a two-stream approach. This does not take account of changes in clouds, stratospheric O_3_ and aerosols, but gives diurnal and seasonal variability. Lightning nitrogen oxide (NO_x_) emissions are calculated within HadGEM2-ES^22^ using a well-established scheme^60^,^61^. This has been re-tuned in comparison with the version quoted in ref. 22, and gives a pre-industrial global source strength of 6.1 TgNyr^−1^.

The pre-industrial and LGM simulations follow the Paleoclimate Model Intercomparison Project 2 (PMIP2) protocols as used in previous work^51^. Land use is not included in either time-period and potential vegetation is simulated dynamically. For the LGM, the model shows a mean tropical cooling of 2.7 °C (consistent with the range of 1.6–3.2 °C in a recent model intercomparison^62^), and an expansion of sea-ice coverage from 5.5 to 7.5% averaged globally. The LGM simulation without interactive chemistry has been evaluated against LGM reconstructions of terrestrial biome distributions, surface temperatures^51^ and dust deposition rates^46^ and reproduces enhanced cooling over land and toward the poles, a reduction in extra-tropical forest coverage and increased dust deposition rates seen in reconstructions. When the chemistry model is employed atmospheric CH_4_ is either allowed to evolve freely (emissions-driven), or is prescribed at the surface (concentration-driven) with values of 675 and 375 p.p.b.v. for the pre-industrial and LGM, respectively.

### Trace gas emissions and soil CH_4_ uptake calculations

The chemistry scheme is driven with monthly varying trace gas emissions from wetlands (CH_4_), biomass burning (CH_4_, CO and NO_x_), vegetation (isoprene and acetone), soil NO_x_, oceans (CH_4_ and CO) and termite and hydrate CH_4_, as well as soil uptake of CH_4_. A summary of the sources and sinks considered is given in Supplementary Table 4.

Wetland CH_4_ emissions are computed within HadGEM2-ES at each model timestep^19^ with a TOPMODEL scheme^21^,^63^,^64^ here configured with a recently derived high-resolution global topographic index data set^65^, which describes the propensity for subgrid areas of soil moisture saturation. Wetland emissions are dependent on soil carbon, soil temperature and the simulated wetland fraction. Soil carbon in HadGEM2-ES is represented by four pools with different turnover rates. The four pools are incremented by litterfall from the dynamic vegetation scheme and are depleted by soil aerobic respiration which is a function of soil temperature and moisture^23^.

Due to the 120 m lowering of sea-level at the LGM, exposed areas of continental shelf can support new wetland areas. The unknown topographic index for these points (currently sub-sea) were calculated using the sub-grid scale topographic variability derived from high-resolution ETOPO1 data set^66^, which has global (land and ocean) coverage. A linear best fit between the observed topographic index field^65^ and the log of orographic roughness performed at 10 arc-minute resolution was used to calculate the topographic index values for exposed areas of continental shelf^51^.

Biomass burning trace gas emissions are applied in HadGEM2-ES following the distribution and magnitude from ref. 67 for the year 1850. The change at the LGM is calculated from simulations with the LPJ-LMfire model^28^. LPJ-LMfire accounts for natural ignition, multi-day burning and coalescence of wildfires. It includes a precipitation dependency of burning versus smouldering, and a representation of human influences. The model was driven with monthly climatologies from HadGEM2-ES combined in an anomaly mode with detrended observational climatologies listed in Table 3 of ref. 28. Land use for the pre-industrial is taken for the year 1770. Two LGM scenarios were run, one without a human influence and a second including a parameterization of the fire activities of hunter-gatherers estimated for this time period^68^.

Termite CH_4_ emissions were calculated on a monthly basis from simulations with the BIOME4 model driven with HadGEM2-ES pre-industrial and LGM climatologies (in an anomaly mode at 0.5° resolution, similar to that described for LPJ-LMfire above). The resultant vegetation distribution was then used with the observed termite biomass per unit area of forest versus grasslands and the observed CH_4_ emissions per unit biomass^69^ to estimate the global emissions. This results in a global pre-industrial source close to 20 TgCH_4_yr^−1^ from independent estimates^3^.

Soil uptake was simulated using the model of ref. 70. This used monthly climatologies of soil temperature (at 10 cm), frozen soil fraction and wetland fraction (where no uptake is allowed) from HadGEM2-ES. Soil types were derived from the Harmonized World Soil Database v1.2 (ref. 71) and were extrapolated to the LGM land-sea-mask.

Acetone and isoprene emissions from plants were simulated using the Joint UK Land Environment Simulator (JULES) version 2.2 (refs 23, 64), which incorporates a biogenic emissions model^72^,^73^. JULES was forced with 3-hourly output from HadGEM2-ES simulations of the pre-industrial and LGM.

The soil NO_x_ model is a semi-empirical scheme^74^, which has been used previously in this context^8^. The ocean BVOC model^31^ was used in the same manner as previous studies^8^, except here we also account for changes in surface ocean dissolved organic carbon between the pre-industrial and LGM by using output from IPSL-CM5A-LR Earth System Model simulations of the pre-industrial and LGM^75^ as archived in the CMIP5 database.

Peatlands CH_4_ emissions are simulated using LPJ-WHyMe^26^, a process-based model of peatland and permafrost processes that includes two peatland plant functional types, vertically resolved production, oxidation and transport of CH_4_ in soil and water table dynamics. The peatland area is prescribed from HadGEM2-ES as the fractional coverage with saturated soil conditions following ref. 76. This results in a pre-industrial peatland CH_4_ flux of 44 TgCH_4_yr^−1^, comparable to other estimates^77^. This is nearly three times larger than the 16 TgCH_4_yr^−1^ simulated with the HadGEM2-ES wetland scheme for an identical wetland area. For consistency when including the peatland fluxes in budget calculations, the non-peatland wetland CH_4_ flux is scaled so that the global pre-industrial wetland plus peatland CH_4_ flux is unchanged.

The remaining trace gas emissions do not change between pre-industrial and LGM simulations, and follow the distributions used in HadGEM2-ES for the CMIP5 pre-industrial simulation^58^, representative of the year 1860. These emissions are C_2_H_6_ (ethane), C_3_H_8_ (propane), HCHO (formaldehyde) and MeCHO (acetaldehyde), where Me=CH_3_. The overall non-CH_4_ trace gas emission budget is summarized in Supplementary Table 1.

### Model simulation set-up

The model simulations consist of three phases (see Supplementary Fig. 1). In phase 1, pre-industrial and LGM HadGEM2-ES simulations were carried out with the atmospheric chemistry deactivated, as reported previously^51^. In these HadGEM2-ES simulations, the soil carbon and vegetation are in equilibrium^51^. The monthly climatologies were then used to force each of the offline trace gas emissions models and the soil CH_4_ uptake model described above.

In phase 2, the physical atmosphere and land surface fields in the coupled chemistry-climate HadGEM2-ES simulations were initialized from the final state of these phase 1 HadGEM2-ES simulations. The atmospheric chemistry trace gases were initialized with fields from a separate pre-industrial simulation with HadGEM2-ES with tropospheric chemistry activated. The climate-chemistry model was then spun up for 100 years using the trace gas emissions and soil CH_4_ uptake calculated with offline models in phase 1. The last 30 years of each chemistry-climate simulation were averaged for analysis.

In phase 3, further sensitivity simulations were branched off from year 50 of the phase 2 simulations. In the sensitivity simulations either (i) lightning, (ii) CO, NO_x_ and BVOCs, or (iii) climate follow pre-industrial conditions but the rest of the model sees LGM conditions. Simulations (i) and (ii) were branched off from the emissions-driven LGM simulation and simulation (iii) was branched off from the pre-industrial simulation. These sensitivity simulations are at least 50 years long, and trends of less than 10 p.p.b.v. per 100 years in global mean surface CH_4_ mixing ratio are taken as steady state, which is satisfied in each case (see Supplementary Fig. 2 and Supplementary Note 1). Prescribed surface CH_4_ mixing ratio (that is, concentration-driven) simulations were also branched off from the dynamic emissions-driven runs in phase 2 and are 30 years long for the offline photolysis and 20 years long when the photolysis rates are interactively calculated within HadGEM2-ES (see Supplementary Note 1).

### Offline CH_4_ concentration calculation

Because of the computational expense of HadGEM2-ES, we use a simplified mass balance calculation of the atmospheric concentration to test the sensitivity to different combinations of total CH_4_ emissions and lifetime changes between the pre-industrial and LGM. For this, we employed the formulation 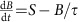, where *B* is the tropospheric CH_4_ burden in Tg, *S* is the global CH_4_ source (TgCH_4_yr^−1^) and 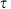 is the tropospheric CH_4_ lifetime in years, and assuming steady state in each time period, that is, 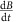
=0. *B* is converted to a volume concentration using a conversion factor *k* which can vary as a function of tropopause height and the distribution of atmospheric CH_4_. HadGEM2-ES predicts that *k* reduces by 2.7% at the LGM, so we include this reduction from *k*=2.6 Tgp.p.b.v.^−1^ for the pre-industrial. 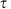 is set to 10.4 years for the pre-industrial as simulated in HadGEM2-ES. The atmospheric concentration for the PI is 660 p.p.b.v. This formulation reproduces the emissions-driven LGM concentration simulated by HadGEM2-ES to within 2%.

A self-feedback factor of *F*=1.26 was calculated from the emissions-driven and concentration-driven lifetime and concentration values (Table 2) following equations 1 and 2 of ref. 78. This is used to augment the LGM [CH_4_] for the source-induced [CH_4_] changes, which otherwise do not include this feedback. Changes in the net source and lifetime are imposed based on the offline trace gas emission simulations and the coupled climate-chemistry simulations (Tables 1 and 2). Hence the self-feedback factor for CH_4_ lifetime is implicitly included. Details of these calculations are given in Supplementary Table 2 and examples are given in Supplementary Note 4.

The required source to reproduce the observed LGM CH_4_ concentration of 375 p.p.b.v. can be calculated by using the lifetime changes for the three scenarios and the equation above. In this case, the constant *k* is reduced by 2.7% for the LGM calculation. This results in *S* values for the LGM of 89, 86 and 85 TgCH_4_yr^−1^ for the low-fire, standard-fire and standard+LGM human scenarios, respectively. Relative to a net source of 165 TgCH_4_yr^−1^ for the pre-industrial (Table 1), these values represent reductions of 46%, 48% and 49%, respectively.

### Data availability

The source code for HadGEM2-ES is part of the UK Met Office Unified Model and is subject to Crown Copyright. The Met Office Unified Model is available for use under licence. For more information see http://www.metoffice.gov.uk/research/collaboration.

The Earth System model variables analysed in the study are available from www.bridge.bris.ac.uk/resources/simulations.

## Additional information

**How to cite this article:** Hopcroft, P. O. *et al*. Understanding the glacial methane cycle. *Nat. Commun.* **8,** 14383 doi: 10.1038/ncomms14383 (2017).

**Publisher's note**: Springer Nature remains neutral with regard to jurisdictional claims in published maps and institutional affiliations.

## Supplementary Material

Supplementary Information Supplementary Figures, Supplementary Tables, Supplementary Notes, Supplementary References.

## Figures and Tables

**Table 1:** Global CH_4_ emissions diagnosed (wetlands and OH and stratospheric loss) or else prescribed within HadGEM2-ES climate-chemistry simulations.

	**PI**	**LGM**		
	**Control**	**Low-fire**	**Standard**	**Standard+LGM humans**
Sources (TgCH_4_yr^−1^)				
Wetlands	138.2	96.8	96.8	96.8
Wetlands+peatlands	138.2	80.5	80.5	80.5
Biomass burning	14.0	1.4	9.2	11.6
Oceans	15.0	12.1	12.1	12.1
Termites	20.0	11.6	11.6	11.6
Hydrates	10.0	0	0	0
SUM	197.2	121.9	129.7	132.1
				
Sinks (TgCH_4_yr^−1^)				
Tropospheric OH loss	160	102	—	—
Soil uptake	11	6	—	—
Added stratospheric loss	21	12	—	—

Atmospheric loss terms are diagnosed within the low-fire emissions-driven simulation for consistency with the emissions listed. The LGM sum excludes the treatment of peatlands and is reduced by 16.3TgCH_4_yr^−1^ when they are considered.

**Table 2:** Global summary of the simulated CH_4_ lifetime with respect to OH 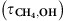 .

	**Pre-Ind**	**LGM**		
	**Control**	**Low-fire**	**Standard**	**+LGM humans**
Emissions-driven				
Offline photolysis	10.4	11.1 (+6.5%)	—	—
Offline photolysis, PI lightning NO_x_	—	9.9	—	—
Offline photolysis, PI non-CH_4_ surf. fluxes	—	13.5	—	—
Offline photolysis, PI climate	—	8.6	—	—
				
Concentration-driven				
Offline photolysis	10.5	10.7 (+2.3%)	11.2 (+6.5%)	11.3 (7.7%)
Fast-J photolysis	7.3	7.3 (−0.2%)	—	—
Fast-J photolysis, +3% strat. O_3_	—	7.4 (+1.7%)	—	—
Fast-J photolysis, Murray *et al*.^16^ strat. O_3_	—	7.4 (+2.1%)	—	—

For the three fire-emissions scenarios: (i) low-fire, (ii) standard (based on LPJ-LMfire) and (iii) standard with LGM human-induced fires at the LGM. The percentage change relative to the respective pre-industrial control simulation is given in brackets.
