# Supplementary material for: Understanding the glacial methane cycle
Source: Nat Commun. 2017 Feb 21;8:14383. doi: 10.1038/ncomms14383 (PMC5321756; doi:10.1038/ncomms14383)
Supplement: Supplementary Information — Supplementary Figures, Supplementary Tables, Supplementary Notes, Supplementary References. [file ncomms14383-s1.pdf]

Supplementary Note 1: Climate-chemistry simulations

The tropospheric methane lifetime with respect to OH calculated from HadGEM2-ES is summarised for the different simulations in table 2 of the main text. The tropospheric CH_4 lifetime was calculated using the simulated monthly mean diagnosed tropopause (1) and the monthly CH_4 and OH mixing ratios following ref. (2).

The timeseries of global mean surface CH_4 mixing ratio and CH_4 growth rate are shown in Supplementary Figure 2. The average trends in ppbv per century (ppbv $100a^{-1}$) averaged over the last 35 years of each simulation are overlain on the lower panels. All simulations are below 10ppbv $100a^{-1}$ and so are judged to be in equilibrium.

Supplementary Note 2: Photolysis and stratospheric O_3

In HadGEM2-ES the photolysis rates are pre-computed(2) and so cannot respond to changes in the radiation balance in the model. Additional simulations with interactively calculated photolysis rates(2) were performed using a version of HadGEM2-ES that includes the Fast-J scheme(3). These simulations are otherwise identical to low-fire simulations described in the main text, including the same trace gas emissions and other boundary conditions. In these simulations the surface CH_4 concentration is prescribed with the global mean values for the pre-industrial or LGM.

Including the dynamic interactive photolysis scheme caused the present-day lifetime to reduce by 28%(2). The difference in lifetime between the PI and LGM simulations was also reduced to a negligible -0.2% compared to a 2.3% increase in the model configuration with pre-calculated photolysis rates. Thus, the impact of photolysis is smaller than the other factors considered.

A further consideration for the photolysis rates is the change in stratospheric ozone (4). HadGEM2-ES does not incorporate stratospheric halogen chemistry processes. For this reason, as noted in the Methods section of the main text, the O_3 field is overwritten by an observationally-based climatology. This is performed from 3 levels above the diagnosed tropopause. To test the role of stratospheric O_3 we additionally prescribed a 3% O_3 increase throughout the stratosphere in a LGM simulation consistent with past work (5; 6). This results in a 2% increase in the LGM CH_4 lifetime relative to the pre-industrial. Thus the lifetime change between the LGM and pre-industrial is 2% in the simulations with pre-computed photolysis and 2% when interactive photolysis rates with a 3% increase in stratospheric O_3 mixing ratios are included.

We performed a further LGM simulation in which the monthly-mean O_3 field at model levels from the tropopause and upwards from the pre-industrial simulation are prescribed in radiation code of the LGM simulation. This removes any difference in stratospheric O_3. These sensitivity tests show little sensitivity to the level at which stratospheric O_3 is prescribed.

We then performed a final test in which the stratospheric O_3 is modified based on the troposphere-stratosphere chemistry simulations for the pre-industrial and LGM from ref. (5). The results show a similar 1.7% increase in lifetime, relative to the pre-industrial. We therefore use this final simulation to quantify the impact of changes in stratospheric O_3 during the LGM in figure 3 of the main text.

Supplementary Note 3: Alternative CH_4 source scenarios

In order to address uncertainty in the make-up of natural CH_4 sources two additional source scenarios are used in offline mass-balance calculations of the LGM CH_4 concentration. These alternative emission scenarios are based on published pre-industrial or modern emission categories as summarised in Supplementary Table 3. Harder07 is based on the Holocene base in ref. (7) and Kirschke13 is based on the average bottom-up estimates for the past three decades (8). Wetland and fire emissions are kept as in the default HadGEM2-ES because wildfire emissions in Harder07 and Kirschke13 are more appropriate for present day conditions, and are therefore likely to be substantially smaller than pre-industrial burning rates, because of anthropogenic fire-suppression activities.

In both cases, the non-wetland sources must be scaled down substantially to close the overall CH_4 budget. For the two scenarios, Harder07 and Kirschke13 bottom-up (BU) the scaling factors are 0.7 and 0.35 respectively, resulting in a global total source of 186TgCH_4yr^{-1} in both cases (and including the sink term of 11.2TgCH_4yr^{-1}). LGM changes are applied to wetlands, fires, oceans, termites and the soil sink as in the standard HadGEM2-ES scenario. Freshwater emissions are scaled with the wetland flux, but no change is applied to the wild animal, permafrost, hydrate or geological source terms, reflecting incomplete knowledge of these systems. The resultant LGM predicted concentration is 479 and 495ppbv (Harder07 and Kirschke13BU, respectively) for a lifetime increase of 6.5% (and including peatland sources). This compares with a value of 464ppbv in the equivalent scenario from Supplementary Table 2. Thus an uncertainty of up to 30ppbv is introduced by considering these alternative representations of the make-up of pre-industrial CH_4 sources.

Supplementary Note 4: Example Offline CH_4 budget calculations

A decrease in emissions due to the LGM wetland change by -41.4 from 165TgCH_4yr^{-1}, gives a fractional source reduction of δ=0.25. This causes a fractional concentration change of $(1+\delta)^F$ (following ref. 9), or $(1\text{-}0.25)^{1.26}$= 0.695. Hence, the LGM concentration change is augmented from -166ppbv to -201ppbv in this case.

The 'net' values in figure 3 and Supplementary Table 2 encompass the range of including peatlands or not, and do not include a reduction in the hydrate term for the LGM (see main text). For the net values, k is reduced by 2.7% at the LGM, as calculated directly from HadGEM2-ES model outputs of tropospheric burden and surface mean mixing ratio. Each net scenario includes a reduction in the stratospheric loss from 21Tgyr^{-1} to 12Tgyr^{-1} as detailed in table 1 of the main text. The self-feedback factor F=1.26 is not included in the sink-driven or net calculations, because the lifetime change from the coupled chemistry-climate model HadGEM2-ES implicitly includes this effect.

In each scenario the atmospheric CH4 concentration $[CH_4]=S \times L/k$. Thus for the pre-industrial, the concentration is $[CH_4]_{PI} = (186-21)\times10.4/2.6$ =660ppbv. For the LGM low-fire with peatlands (a net surface source reduction of -76TgCH4yr^{-1}), the calculated $\Delta[CH_4]_{LGM-PI} = (186-12-76)\times(10.4\times1.023)/(2.6\times0.973)-660$ =-248ppbv. For the LGM with humans

fire and no peatlands (a net surface source reduction of -49.4$TgCH_4yr^{-1}$), $\Delta[CH_4]_{LGM-PI} = (186 - 12 - 49.4) \times (10.4 \times 1.077)/(2.6 \times 0.973) - 660 =$-108ppbv.

Supplementary Table 1: Global non-CH_4 trace gas emissions used in HadGEM2-ES climate-chemistry simulations. Values in brackets include the simulated contribution of human fires at the LGM. Low-fire emissions (not listed) are equal to 10% of pre-industrial values. Units are Tg[species]yr^{-1}, except for NO_x which are TgNyr^{-1}.

	PI control	LGM LPJ-fire (+humans)	LGM/PI (%) LPJ-fire (+humans)
**Vegetation**			
Isoprene	657.0	529.0	81
Acetone	40.0	26.7	67
**Biomass burning**			
CO	322.0	219.2(285.3)	68(89)
Acetone	5.0	3.3(4.3)	66(85)
NOx	4.0	2.8(3.7)	70(93)
**Soils**			
NOx	5.6	7.6	137
**Oceans**			
CO	45	36.5	81
**Lightning**			
NOx	6.1	4.6	75

Supplementary Table 2: Calculations used to produce figure 3 in the main text.

	ΔS ($TgCH_4yr^{-1}$)	$\Delta\ \tau$ (%)	$[CH_4]$(LGM) (ppbv)	$\Delta[CH_4]$ (ppbv)
**Surface processes**				
Wetlands	-41.4	-	459	-201
Wetlands+peatlands	-57.7	-	384	-276
Biomass burning				
1. Low-fire	-12.6		597	-63
2. Standard	-4.7	-	636	-24
3. +LGM humans	-2.3	-	648	-12
Oceans	-2.9	-	645	-15
Termites	-8.4	-	618	-42
Hydrates	-10.0, 0	-	610, 660	-50, 0
Soil uptake	5.6	-	688	28
**Atmospheric chemistry**				
Lightning NO_x	-	6.7	704	44
Non-CH_4 fluxes	-	-25.9	489	-171
Temperature/humidity	-	19.3	787	127
Stratospheric O_3	-	2.1	674	14
**Net**				
Low-fire	-76.0,-59.7	2.3	412, 481	-248, -179
Standard-fire	-68.1,-51.8	6.5	464, 535	-196, -125
+LGM humans	-65.7,-49.4	7.7	480, 552	-181, -108

Supplementary Table 3: Pre-industrial or present day CH_4 emissions in this work and previous studies.

**Sources** ($TgCH_4yr^{-1}$)	This work	V05	H07	K02-06	K13 Bottom-up	K13 Top-down
Wetlands (% total)	138 (70%)	148 (74%)	163 (70%)	110 (61%)	216 (62%)	164 (82%)
Non-wetlands	59	51	69	69	130	37
Freshwater	-	-	5	-	40	-
Wild animals	-	-	19	-	15	-
Wild fires	14.0	11	4	-	3	-
Termites	20.0	27	20	-	11	-
Oceans	15.0	13	13.5	-	1	-
Geological	-	-	7	-	53	-
Hydrates	10.0	-	-	-	6	-
Permafrost	-	-	-	-	1	-
Soil sink*	11.2	-	11.5	14	28	27
Net Sum	186	199	220	165	318	174

VO5: Valdes *et al.,* (2005)[10], K02-06: Kaplan (2002) & Kaplan *et al.,* (2006)[11; 12], H07: Harder*et al.,* (2007)[7], K13: Kirschke *et al.,* (2013)[8].

Supplementary Table 4: Processes affecting the sources and sinks of CH_4 and their level of inclusion in this study.

	HadGEM2-ES Fully interactive	Offline Process	Empirical	Scheme employed
**Sources**				
Wetlands	Y			Gedney *et al.*, 2004[13]
Peatlands		Y		Wania *et al.*, 2010[14]
Biomass burning		Y		Pfeiffer *et al.*, 2013[15]
Termites			Y	This study
Oceans		Y		Guenther *et al.*, 1995[16]
Hydrates				
**Sinks**				
OH oxidation	Y			O'Connor *et al.*, 2014[2]
Soil uptake		Y		Curry, 2007[17]
Stratospheric loss	Y			O'Connor *et al.*, 2014[2]
**Other processes**				
Plant BVOCs (isoprene, acetone)		Y		Pacifico *et al.*, 2011[18]
Ocean VOCs (CO)		Y		Guenther *et al.*, 1995[16]
Biomass burning (CO, NO_x, acetone)		Y		Pfeiffer *et al.*, 2013[15]
Soil NO_x		Y		Yienger & Levy 1995[19]
Lightning NO_x	Y			Price & Rind, 1992,1994[20; 21]
Photolysis	Sensitivity simulations			Wild *et al.*, 2000[3]
Stratospheric O_3	Sensitivity simulations			from Murray *et al.*, 2014[5]

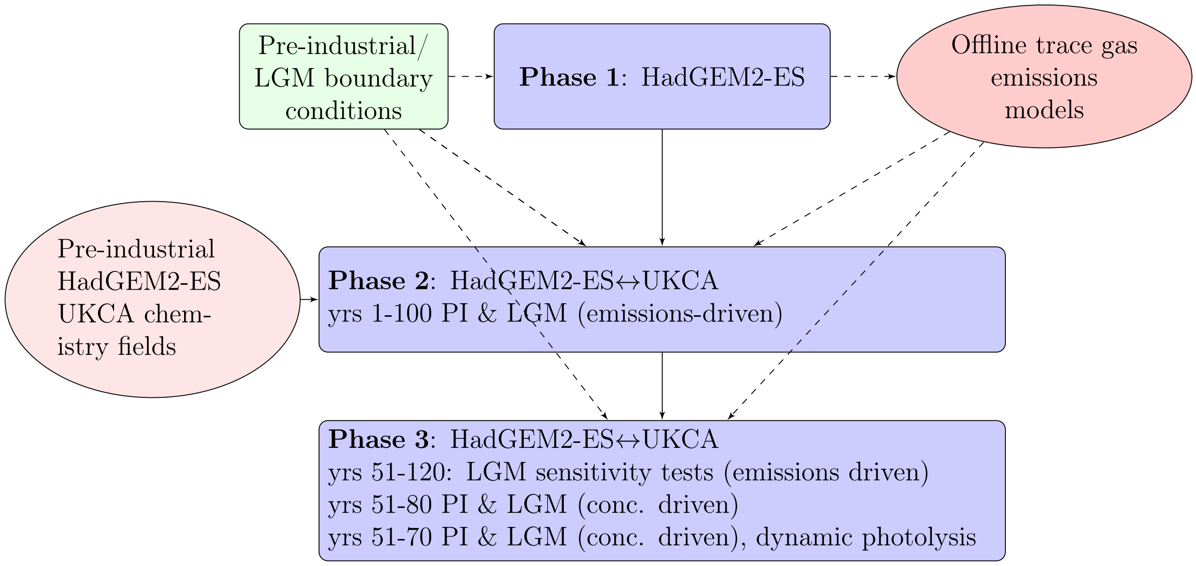

Supplementary Figure 1: Flow chart showing model simulation setup. HadGEM2-ES: atmosphere-land surface-aerosols. HadGEM2-ES↔UKCA: atmosphere-land surface-aerosols-chemistry. Offline models are: JULES (BVOCs), LPJ-LMfire (biomass burning), Yienger & Levy (1995) (soil NO_x), Guenther *et al* (1995) (ocean BVOCs), LPJ-WHyMe (peatland CH_4), Curry (2007) (soil CH_4 uptake) and this work (termite CH_4).

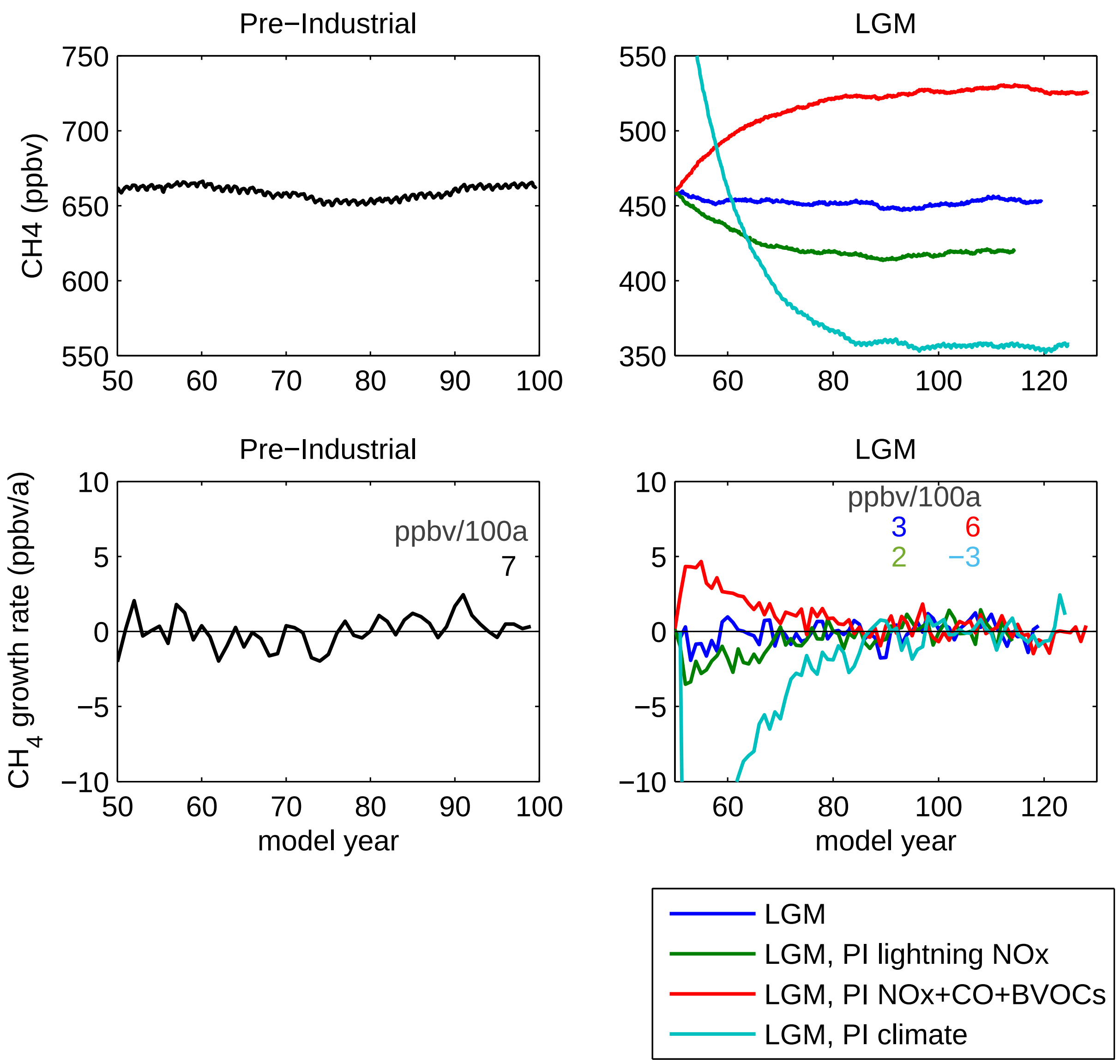

Supplementary Figure 2: CH_4 concentration (top row) and concentration growth rate (ppbvyr^{-1}) (bottom row) in each emissions-driven HadGEM2-ES simulation, excluding 50 years of initial spinup. The numbers overlain show the mean growth rate per 100 years calculated over the final 35 years of each simulation. Values less than 10ppbv 100a^{-1} are deemed to be in equilibrium.

References

[1] Hoerling, M., Schaack, T. & Lenzen, A. A global analysis of stratosphere-tropospheric exchange during northern winter. *Mon Weather Rev* **121**, 162–172 (1993).

[2] O'Connor, F. *et al.* Evaluation of the new UKCA climate-composition model - Part 2: The Troposphere. *Geosci. Model Dev.* 41–91 (2014).

[3] Wild, O., Zhu, X. & Prather, M. Fast-J: Accurate simulation of in- and below-cloud photolysis in tropospheric chemical models. *Atmo Chem* **37**, 245–282 (2000).

[4] Bekki, S., Law, K. & Pyle, J. Effect of ozone depletion on atmospheric CH_4 and CO concentrations. *Nature* **371**, 595–597 (1994).

[5] Murray, L. *et al.* Factors controlling variability in the oxidative capacity of the troposphere since the Last Glacial Maximum. *Atmos Chem Phys* **14**, 3589–3622 (2014).

[6] Rind, D., Lerner, J., McLinden, C. & Perlwitz, J. Stratospheric ozone during the Last Glacial Maximum. *Geophys Res Lett* **36** (2009).

[7] Harder, S. *et al.* A global climate model study of CH4 emissions during the Holocene and glacial-interglacial transitions constrained by ice core data. *Glob Biogeochem Cyc* **21**, GB1011 (2007).

[8] Kirschke, S. *et al.* Three decades of global methane sources and sinks. *Nature Geosci* **6**, 813–822 (2013).

[9] O'Connor, F. *et al.* Possible role of wetlands, permafrost and methane hydrates in the methane cycle under future climate change: a review. *Rev Geophys* **48**, RG4005 (2010).

[10] Valdes, P., Beerling, D. & Johnson, C. The ice age methane budget. *Geophys. Res. Lett.* **32** (2005).

[11] Kaplan, J. Wetlands at the Last Glacial Maximum: Distribution and methane emissions. *Geophys. Res. Lett.* **29** (2002).

[12] Kaplan, J., Folberth, G. & Hauglustaine, D. Role of methane and biogenic volatile organic compound sources in the late glacial and Holocene fluctuations of atmospheric methane concentrations. *Glob. Biogeochem. Cyc.* **20**, GB2016 (2006).

[13] Gedney, N., Cox, P. & Huntingford, C. Climate feedback from wetland methane emissions. *Geophys. Res. Lett.* **31** (2004).

[14] Wania, R., Ross, I. & Prentice, I. C. Implementation and evaluation of a new methane model within a dynamic global vegetation model: LPJ-WHyMe v1.3.1. *Geoscientific Model Development* **3** (2010).

[15] Pfeiffer, M., Spessa, A. & Kaplan, J. A model for global biomass burning in preindustrial time: LPJ-LMfire (v1.0). *Geosci Model Dev* 643–685 (2013).

[16] Guenther, A. *et al.* A global model of natural volatile organic compound emissions. *J. Geophys. Res.* **100**, 8873–8892 (1995).

[17] Curry, C. Modeling the soil consumption of atmospheric methane at the global scale. *Global Biogeochem. Cycles* **21** (2007).

[18] Pacifico, F. *et al.* Evaluation of a photosynthesis-based biogenic isoprene emission scheme in JULES and simulation of isoprene emissions under present-day climate conditions. *Atmos Chem Phys* **11**, 4371–4389 (2011).

[19] Yienger, J. & Levy II, H. Empirical model of global soil-biogenic NO_x emissions. *J Geophys Res* **100**, 11447–11464 (1995).

[20] Price, C. & Rind, D. A simple lightning parameterization for calculating global lightning distributions. *J Geophys Res* **92**, 9919–9933 (1992).

[21] Price, C. & Rind, D. Modeling global lightning distributions in a general circulation model. *Mon. Weather Rev.* **122**, 1930–1939 (1994).
